# Emphysematous Pyelonephritis in a Non-diabetic Patient Presenting With Septic Shock: A Report of a Rare Case

**DOI:** 10.7759/cureus.102167

**Published:** 2026-01-23

**Authors:** Anas E Ahmed, Lama H Bin Saeed, Mohammed M Alsharif, Mojtaba M Syyab, Omar M Alotaibi

**Affiliations:** 1 Community Medicine, Jazan University, Jazan, SAU; 2 College of Medicine, King Saud bin Abdulaziz University for Health Sciences, Riyadh, SAU; 3 General Practice, King Khalid Hospital, Tabuk, SAU; 4 College of Medicine, King Abdulaziz University, Jeddah, SAU; 5 College of Medicine, Taif University, Taif, SAU

**Keywords:** computed tomography, emphysematous pyelonephritis, gas-forming renal infection, nephrectomy, non-diabetic patient, septic shock

## Abstract

Emphysematous pyelonephritis is a rare and fulminant necrotizing infection of the kidney characterized by gas formation within the renal parenchyma and surrounding tissues. It is traditionally associated with diabetes mellitus and carries significant morbidity and mortality if diagnosis and management are delayed. Occurrence in non-diabetic patients is uncommon and may pose diagnostic challenges due to a lower index of clinical suspicion.

We report the case of a 56-year-old non-diabetic man who presented with an acute onset of left flank pain, high-grade fever, and systemic toxicity. Clinical examination revealed features of sepsis, and laboratory investigations demonstrated leukocytosis, markedly elevated inflammatory markers, and acute kidney injury, with normal glycemic indices. Imaging studies played a pivotal role in diagnosis, with plain radiography and ultrasonography suggesting intrarenal gas, and contrast-enhanced computed tomography confirming extensive gas within the renal parenchyma and perinephric space, consistent with emphysematous pyelonephritis. Despite aggressive resuscitation and broad-spectrum intravenous antibiotics, the severity of renal involvement and hemodynamic instability necessitated emergency nephrectomy. The patient had a complicated but ultimately favorable postoperative course with recovery of renal function and resolution of sepsis. This case emphasizes that emphysematous pyelonephritis can occur in the absence of diabetes mellitus and may follow an aggressive clinical course. Early recognition, prompt cross-sectional imaging, and individualized management guided by disease severity are crucial to improving outcomes. Clinicians should maintain a high degree of suspicion for emphysematous pyelonephritis in patients presenting with severe upper urinary tract infection and sepsis, regardless of diabetic status.

## Introduction

Emphysematous pyelonephritis is a rare, severe, and potentially life-threatening necrotizing infection of the renal parenchyma and surrounding tissues, characterized by the presence of gas within the renal system [1]. It represents a urological emergency with historically high mortality rates if not promptly recognized and managed [1,2]. The condition is most commonly associated with uncontrolled diabetes mellitus, which is present in the majority of reported cases, and is thought to predispose patients through impaired immunity, high tissue glucose concentrations, and microvascular compromise [2,3]. Other recognized risk factors include urinary tract obstruction, urolithiasis, immunosuppression, and chronic kidney disease [2]. Gas-forming organisms, most frequently *Escherichia coli *and *Klebsiella pneumoniae*, are typically implicated. Advances in imaging modalities, particularly computed tomography, have significantly improved diagnostic accuracy and facilitated early intervention [1-4].

Although emphysematous pyelonephritis predominantly affects diabetic individuals, its occurrence in non-diabetic patients is uncommon and not well-characterized in the literature [1-4]. In such cases, diagnosis may be delayed due to a lower index of clinical suspicion, potentially leading to worse outcomes [2,4]. The pathophysiological mechanisms in non-diabetic patients remain poorly understood but are believed to involve local tissue ischemia, impaired host defense, and infection with highly virulent organisms [1,3]. Reporting cases of emphysematous pyelonephritis in non-diabetic patients is therefore important to expand current understanding of its clinical spectrum, highlight diagnostic challenges, and emphasize the need for prompt imaging and aggressive management regardless of glycemic status.

## Case presentation

A 56-year-old man presented to the emergency department with a 5-day history of progressively worsening left flank pain associated with high-grade fever, chills, nausea, and reduced oral intake. The pain was constant, severe, radiating to the left lower abdomen, and partially relieved by analgesics. He also reported dysuria and increased urinary frequency for one week, but denied hematuria, pneumaturia, vomiting, bowel disturbances, or recent trauma. There was no history of diabetes mellitus, hypertension, chronic kidney disease, urolithiasis, or immunosuppressive therapy. He had no prior hospitalizations or urological interventions. The patient was a non-smoker, consumed alcohol occasionally, and had no known drug allergies. Family history was unremarkable for metabolic or renal diseases.

On arrival, the patient appeared ill and toxic. Vital signs revealed a temperature of 39.2 °C, heart rate of 112 beats per minute, blood pressure of 96/58 mmHg, respiratory rate of 22 breaths per minute, and oxygen saturation of 97% on room air. He was alert and oriented but visibly uncomfortable. General examination showed mild pallor and signs of dehydration. Cardiovascular and respiratory examinations were unremarkable. Abdominal examination revealed tenderness over the left flank and left costovertebral angle with guarding but no palpable mass. The abdomen was soft with normal bowel sounds and no organomegaly. Digital rectal examination was normal, and there was no peripheral edema or skin crepitus.

Initial laboratory investigations demonstrated leukocytosis with a white blood cell count of 18.6 × 10⁹ per liter (neutrophils 89 percent), hemoglobin of 11.8 grams per deciliter, and platelet count of 240 × 10⁹ per liter. Serum inflammatory markers were elevated, with C-reactive protein of 186 milligrams per liter and procalcitonin of 12 nanograms per milliliter. Renal function tests showed acute kidney injury with serum creatinine of 2.1 milligrams per deciliter (baseline unknown) and blood urea nitrogen of 48 milligrams per deciliter. Serum electrolytes revealed mild hyponatremia (130 millimoles per liter) and normal potassium levels. Liver function tests were within normal limits. Random blood glucose was 98 milligrams per deciliter, and glycated hemoglobin of 5.4% confirmed the absence of diabetes mellitus. Urinalysis showed turbid urine with positive leukocyte esterase, nitrites, numerous pus cells, and moderate bacteriuria. Urine culture later grew *Escherichia coli* sensitive to carbapenems and third-generation cephalosporins. Blood cultures were positive for the same organism (Table 1).

**Table 1:** Summary of laboratory investigations at presentation and during diagnostic workup Reference ranges correspond to standard adult values. Abbreviations: HPF, high-power field

Laboratory Investigation	Patient Value	Unit	Reference Range
White blood cell count	18.6	×10⁹/L	4.0–11.0
Neutrophils	89	%	40–75
Hemoglobin	11.8	g/dL	13.0–17.0
Hematocrit	35	%	40–50
Platelet count	240	×10⁹/L	150–400
C-reactive protein	186	mg/L	<5
Procalcitonin	12	ng/mL	<0.05
Serum creatinine	2.1	mg/dL	0.7–1.3
Blood urea nitrogen	48	mg/dL	7–20
Sodium	130	mmol/L	135–145
Potassium	4.2	mmol/L	3.5–5.1
Chloride	98	mmol/L	98–107
Bicarbonate	20	mmol/L	22–29
Aspartate aminotransferase	32	U/L	10–40
Alanine aminotransferase	28	U/L	7–56
Alkaline phosphatase	96	U/L	44–147
Total bilirubin	0.8	mg/dL	0.3–1.2
Serum albumin	3.1	g/dL	3.5–5.0
Random blood glucose	98	mg/dL	70–140
HbA1c	5.4	%	<5.7
Appearance	Turbid	—	Clear
Leukocyte esterase	Positive	—	Negative
Nitrites	Positive	—	Negative
White blood cells	Numerous	/HPF	0–5
Red blood cells	2–3	/HPF	0–2
Bacteria	Moderate	—	None
Protein	Trace	—	Negative
Urine culture	Escherichia coli	—	No growth
Blood culture	Escherichia coli	—	No growth
Antibiotic sensitivity	Sensitive to carbapenems and third-generation cephalosporins	—	—

**Figure 1:**
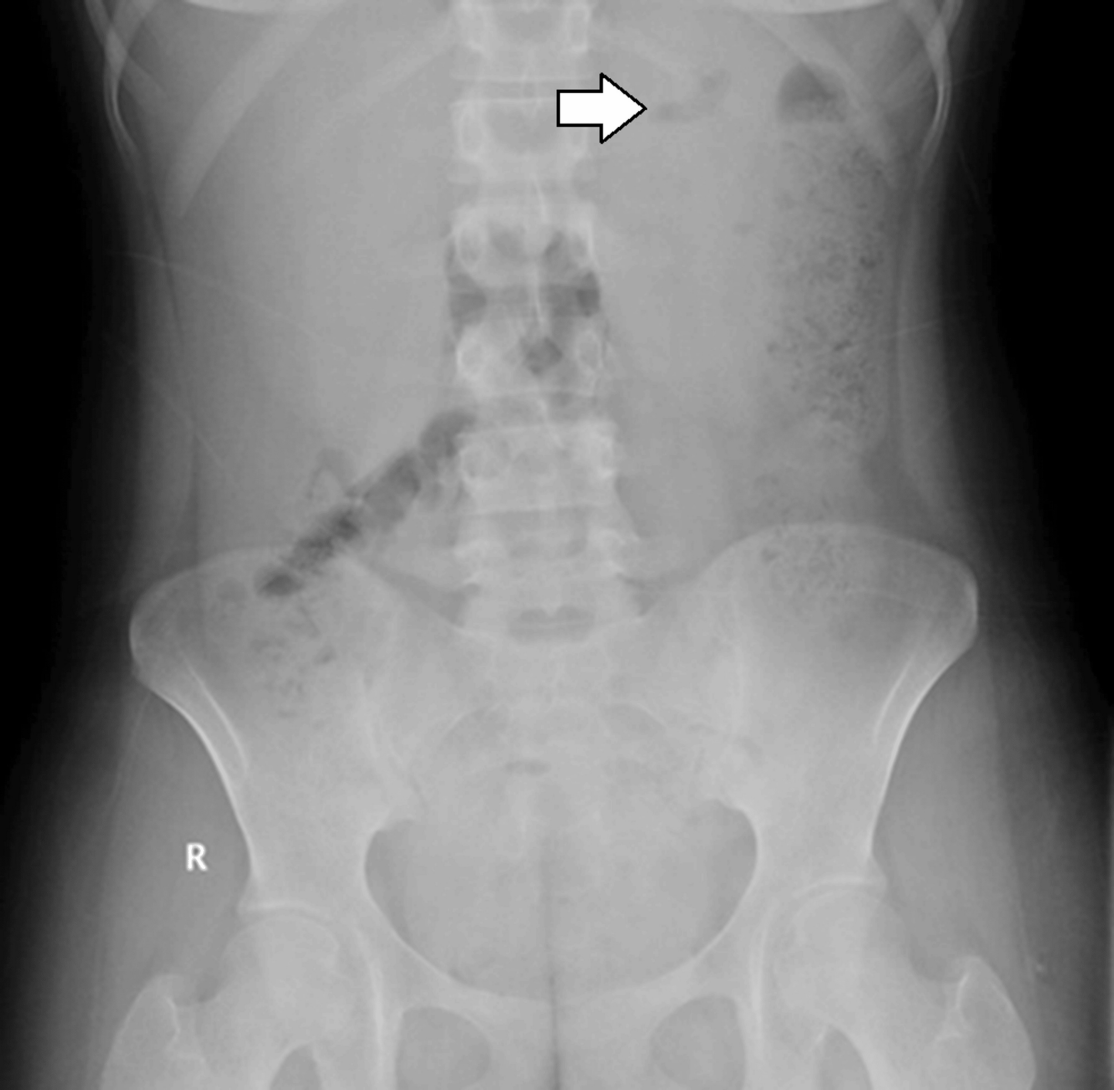
Abdominal radiograph of the left kidney region Plain abdominal radiograph showing abnormal air lucency projected over the left kidney (arrow). This finding is suggestive but not diagnostic of intrarenal gas.

**Figure 2:**
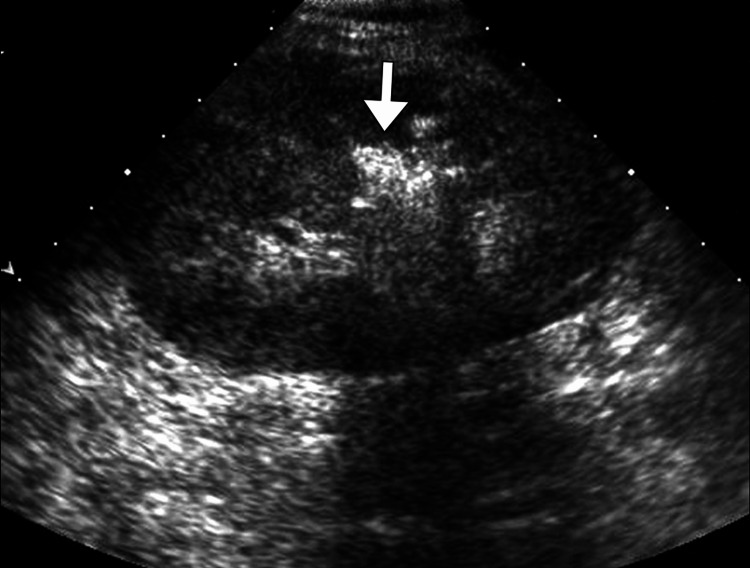
Ultrasound of the left kidney Grayscale ultrasound image showing an echogenic focus (arrow) within the renal parenchyma with posterior acoustic shadowing, representing intrarenal gas. Ultrasound may raise suspicion for emphysematous pyelonephritis, but it is not definitive.

**Figure 3:**
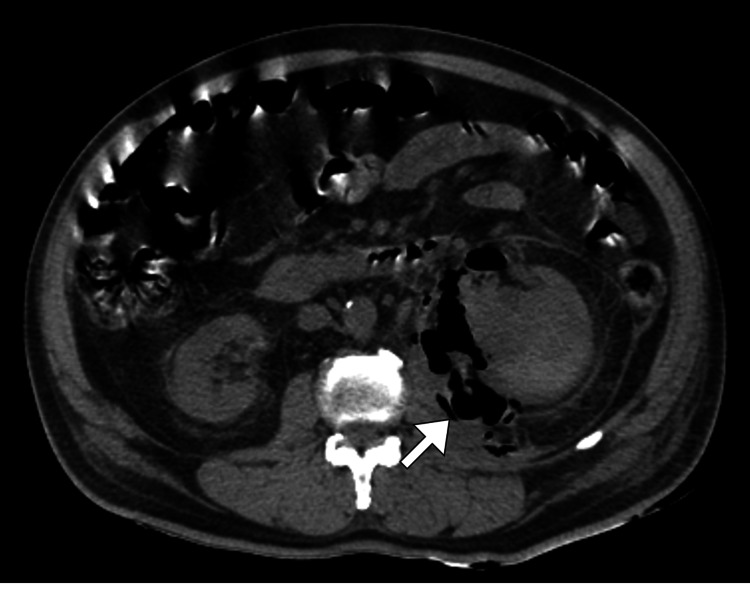
Computed tomography confirming emphysematous pyelonephritis Axial computed tomography (CT) image showing a hypodense collection posterior to the left kidney (arrow) containing multiple foci of gas. CT is the gold standard for diagnosis, accurately demonstrating the presence, extent, and distribution of gas in emphysematous pyelonephritis.

An initial plain abdominal radiograph demonstrated mottled areas of radiolucency over the left renal region, suggestive of gas (Figure 1). Ultrasonography of the abdomen revealed an enlarged left kidney with increased cortical echogenicity, poor corticomedullary differentiation, and multiple echogenic foci with posterior dirty shadowing consistent with intrarenal gas (Figure 2). Mild perinephric fluid collection was also noted. Given the high suspicion of emphysematous infection, a contrast-enhanced computed tomography scan of the abdomen and pelvis was performed. Computed tomography imaging confirmed the presence of extensive gas within the left renal parenchyma and collecting system with extension into the perinephric space, without evidence of obstructing calculi (Figure 3). There was associated renal parenchymal destruction but no distant gas or abscess formation. The right kidney appeared normal. Despite the absence of diabetes mellitus, the diagnosis of emphysematous pyelonephritis was established due to characteristic radiological features and septic presentation.

The patient was admitted to the intensive care unit for close monitoring and management. Initial treatment included aggressive intravenous fluid resuscitation, vasopressor support for septic shock, and empiric broad-spectrum intravenous antibiotics with meropenem, later tailored according to culture sensitivity results. A multidisciplinary team involving urology, radiology, nephrology, and critical care specialists was consulted. Given the extensive renal involvement and clinical instability, conservative management with antibiotics alone was deemed insufficient. Percutaneous drainage was considered; however, due to diffuse parenchymal destruction and poor renal function, the patient underwent an emergency left nephrectomy.

The postoperative course was complicated by transient hypotension requiring continued vasopressor support and acute kidney injury, which gradually improved with supportive care. The patient was weaned off vasopressors by postoperative day three and transferred to the general ward on day five. Renal function stabilized with serum creatinine decreasing to 1.4 milligrams per deciliter. He completed a 14-day course of intravenous antibiotics followed by oral therapy.

At follow-up four weeks after discharge, the patient was clinically stable, afebrile, and asymptomatic. Laboratory tests showed normalized inflammatory markers and stable renal function. He was counseled regarding long-term follow-up and lifestyle measures, and no recurrence or complications were noted during subsequent outpatient visits.

## Discussion

Emphysematous pyelonephritis is an uncommon but fulminant necrotizing infection of the kidney characterized by gas formation within the renal parenchyma, collecting system, or perinephric tissues [1,2]. Since its first description, emphysematous pyelonephritis has been recognized as a urological emergency due to its rapid progression to septic shock and multiorgan dysfunction if not promptly treated [2,5]. The majority of reported cases occur in patients with diabetes mellitus, accounting for up to 80-90% of cases in large series, which has shaped the traditional perception of emphysematous pyelonephritis as a predominantly diabetic complication [4-7]. The present case is therefore notable, as it underscores that emphysematous pyelonephritis can develop in non-diabetic individuals and may follow an equally aggressive clinical course, reinforcing the need for heightened clinical vigilance irrespective of glycemic status.

The pathogenesis of emphysematous pyelonephritis is multifactorial and incompletely understood. In diabetic patients, high tissue glucose levels facilitate rapid fermentation by gas-forming organisms, while impaired host immunity and microvascular disease contribute to tissue ischemia and necrosis [1-7]. In non-diabetic patients, alternative mechanisms have been proposed, including local tissue hypoxia, impaired renal perfusion, urinary tract obstruction, and infection with highly virulent organisms capable of gas production even in the absence of hyperglycemia [2,5]. Acute inflammation, vascular thrombosis, and parenchymal infarction may create a low-oxygen environment conducive to anaerobic metabolism and gas formation [3,5]. In the current case, no overt obstruction or metabolic abnormality was identified, suggesting that host factors and organism virulence may play a dominant role in disease development.

*Escherichia coli* remains the most frequently isolated pathogen in emphysematous pyelonephritis, followed by *Klebsiella pneumoniae*, *Proteus *species, and, less commonly, anaerobes and fungi [3-6]. The isolation of *Escherichia coli *from both urine and blood cultures in this patient is consistent with existing literature and supports hematogenous dissemination as a contributor to systemic sepsis [3,4]. Antimicrobial resistance patterns are increasingly relevant, as delayed initiation of appropriate antibiotics has been associated with poor outcomes. Early empiric broad-spectrum antimicrobial therapy, followed by culture-directed treatment, is therefore essential in suspected cases [1-7].

Imaging plays a pivotal role in the diagnosis, classification, and management planning of emphysematous pyelonephritis [1-4]. Plain radiography and ultrasonography may suggest the diagnosis by demonstrating gas within the renal region; however, computed tomography remains the gold standard due to its superior sensitivity and ability to delineate the extent of gas, parenchymal destruction, and extrarenal spread [3,6]. Computed tomography-based classification systems have prognostic value and help guide therapeutic decisions, with more extensive disease correlating with higher morbidity and mortality. In non-diabetic patients, where clinical suspicion may be lower, early computed tomography imaging is particularly crucial to avoid diagnostic delay [2-5].

Management strategies for emphysematous pyelonephritis have evolved significantly over the past two decades [1,6]. Historically, emergent nephrectomy was considered the standard of care, given the high mortality associated with conservative treatment [2,3]. With advances in imaging, critical care, and interventional radiology, a more conservative, kidney-preserving approach combining antibiotics with percutaneous drainage has gained acceptance in selected patients [3,6]. Nevertheless, surgical intervention remains indispensable in cases with extensive parenchymal destruction, hemodynamic instability, or failure of conservative measures. The need for nephrectomy in the present case reflects the severity of infection and highlights that non-diabetic status does not necessarily predict a milder disease course [1,7].

## Conclusions

This case highlights that emphysematous pyelonephritis, although classically associated with diabetes mellitus, can occur in non-diabetic patients and may present with rapidly progressive sepsis and acute kidney injury. A high index of suspicion, prompt laboratory evaluation, and early use of cross-sectional imaging, particularly computed tomography, are critical for timely diagnosis. Management should be individualized and guided by clinical severity and radiological extent, with early multidisciplinary involvement to optimize outcomes. Aggressive antimicrobial therapy, close hemodynamic monitoring, and timely surgical intervention remain the cornerstones of treatment in severe cases. The absence of diabetes mellitus should not delay consideration of emphysematous pyelonephritis in patients with severe upper urinary tract infection, as early recognition and decisive management are essential to reduce morbidity and mortality.
